# Data on green tea flavor determinantes as affected by cultivars and manufacturing processes

**DOI:** 10.1016/j.dib.2016.12.025

**Published:** 2016-12-21

**Authors:** Zhuo-Xiao Han, Mohammad M. Rana, Guo-Feng Liu, Ming-Jun Gao, Da-Xiang Li, Fu-Guang Wu, Xin-Bao Li, Xiao-Chun Wan, Shu Wei

**Affiliations:** a State Key Laboratory of Tea Plant Biology and Utilization, Anhui Agricultural University, 130 Changjiang Ave W., Hefei, Anhui 230036, China; b Bangladesh Tea Research Institute, Srimangal 3210, Moulvibazar, Bangladesh; c Agriculture and Agri-Food Canada, Saskatoon Research and Development Centre, Saskatoon, Saskatchewan, Canada S7N 0X2; d Tea Company of September 16, Shucheng, Anhui 231334, China

## Abstract

This paper presents data related to an article entitled “Green tea flavor determinants and their changes over manufacturing processes” (Han et al., 2016) [1]. Green tea samples were prepared with steaming and pan firing treatments from the tender leaves of tea cultivars ‘Bai-Sang Cha’ (‘BAS’) and ‘Fuding-Dabai Cha’ (‘FUD’). Aroma compounds from the tea infusions were detected and quantified using HS-SPME coupled with GC/MS. Sensory evaluation was also made for characteristic tea flavor. The data shows the abundances of the detected aroma compounds, their threshold values and odor characteristics in the two differently processed tea samples as well as two different cultivars.

**Specifications Table**

**Table:** 

Subject area	Chemistry
More specific subject area	Aroma
Type of data	Table
How data was acquired	HS-SPME coupled with GC/MS
Data format	Analyzed
Experimental factors	Green tea samples were prepared from the fresh leaves of two cultivars following two different processing technology. Then the infusions were prepared brewing the sample leaves in the hot water for 5 min.
Experimental features	Volatile aroma compounds present in the tea infusions were identified and quantified using HS-SPME coupled with GC–MS.
Data source location	Shucheng, Anhui, China (31°31′ 87″ N, 117°02′ 84″ E)
Data accessibility	Data is available with this article

**Value of the data**

• This adds to the limited public datasets available to compare the aroma compounds between the infusions prepared from differently processed green teas as well as from different cultivars.
• Threshold values and odor characteristics of detected volatiles will allow researchers to compare their data independently.
• Standard curves established using authentic compounds can be used by other researchers to quantify the volatiles.
• The data provides information about the changes specific to processing technology and cultivar differences.

## 1 Data

The data presented in Table 1, Table 2, Table 3, Table 4, Table 5, Table 6 display standard curves for compound quantification, tea sensory evaluation results, aroma compounds with varied abundances, perception threshold values in the infusions of the steamed processed (St) and pan firing processed (Pa) teas from cultivars ‘BAS’ and ‘FUD’. Dynamic changes in the abundance of different flavor compounds due to the processing treatments and cultivars can be found in the associated article [1].

## 2 Experimental design, materials and methods

### 2.1 Chemicals

For volatile profiling, authentic standards of linalool, linalool oxides, geraniol, citral, β-myrcene, limonene, β-ocimene, nerol, *trans*-nerolidol, farnesene, β-ionone, geranyl acetone, naphthalene, *cis*-3-hexen-1-ol, nonanal, benzene acetaldehyde, methyl salicylate, *cis*-hexenyl acetate, methyl jasmonate, *cis*-3-hexenyl hexanoate, 3-octen-1-ol, indole and ethyl decanoate were purchased from Sigma-Aldrich (Shanghai, China). *cis*-Jasmone was purchased from Aladdin Industrial Inc. (Shanghai, China).

### 2.2 Volatile profiling

Tea infusions were prepared using the fresh leaf samples and final product tea samples from two cultivars. Volatile collection, identification and quantification were conducted according to Wang et al. [7] using headspace-solid phase micro-extraction (HS-SPME) coupled with gas chromatography (Agilent 7697A)/mass spectrometry (Agilent 7890A) (GC/MS) with some minor modifications. In our experiments, 5 mL tea infusion was used for headspace volatile collection with the fiber (65 μM PDMS/DVB, Supelco, Bellefonte PA, USA) for 1 h. DB-5 capillary column (30 m×0.25 mm×0.25 µm, Agilent) was used for GC/MS analysis. The assays were carried out in triplicate for each sample. Ethyl decanoate (0.01%, 10 μL) was added to the samples as the internal standard. Chemicals were identified by comparing with either the standard substance or the NIST database [8]. Compounds quantification were calculated based either on the calibration curves established using series diluted solutions prepared with authentic standards or on the peak areas of the internal standard. The concentrations of the volatiles were expressed as μg kg^−1^ DW.

### 2.3 Sensory evaluation of tea infusions

Three grams (accurate to 0.01 g) of the processed tea was infused with 150 mL of distilled boiling water for 5 min. By using a sieve, infused leaves were removed and tea infusions were transferred to glasses. The sensory evaluation was carried out by five trained panelists. They were instructed to evaluate the sensory responses regarding taste, aroma, and overall flavor quality by giving a score within 100 and also to note down the flavor characteristics of the samples. Subsequent analyses of the samples were performed in triplicate.The order of the samples was randomized. Between the tastes of the samples, every panelist drank natural mineral water and ate unsalted cracker to vanish the taste. Final sensory scores were statistically analyzed using *T*-test (*P*<0.05).

## Figures and Tables

**Table 1:** Standard curves for the major volatiles established using a series of diluted solutions of authentic compounds.

**Compounds**	**Formula**^a^	***R*^2^**	**Linear range (µg kg**^**−1**^**)**
β-Mycene	*Y*=3E−6X+0.3179	0.9960	2.5–10.0
Limonene	*Y*=8E−6X+0.6167	0.9960	2.5–10.0
β-Ocimene	*Y*=2E−6X−0.2975	0.9959	2.5–10.0
Linalool oxides I	*Y*=2E−6X−0.0786	0.9981	5.0–20.0
Linalool oxides II	*Y*=5E−6X−0.0942	0.9954	5.0–20.0
Linalool	*Y*=4E−6X+2.8332	0.9973	10.0–30.0
Nerol	*Y*=4E−6X−0.4882	0.9999	2.5–10.0
Geraniol	*Y*=3E−6X+5.4386	0.9881	125.0–500.0
Citral	*Y*=4E−6X−1.1757	0.9610	2.5–10.0
Geranyl acetone	*Y*=4E−6X+0.2909	0.9976	2.5–10.0
β-Ionone	*Y*=4E−6X−1.2909	0.9992	5.0–20.0
*trans*-Nerolidol^b^	*Y*=3E−6X+2.1929	0.9881	5.0–20.0
β-Farnesene	*Y*=5E−6X+0.0771	0.9976	2.5–10.0
Methyl salicylate	*Y*=4E−6X−2.1275	0.9999	5.0–20.0
*cis*-3-Hexenyl hexanoate	*Y*=1E−6X+0.8646	0.9865	2.5–10.0
Methyl jasmonate	*Y*=2E−6X−0.6155	0.9999	2.5–10.0
*cis*-Hexenyl acetate	*Y*=9E−6X+5.1199	0.9728	10.0–30.0
Nonanal	*Y*=9E−6X+6.6491	0.9728	10.0–30.0
*cis*-3-hexen-1-ol	*Y*=4E−6X+1.8449	0.9534	2.5–10.0
3-Octen-1-ol	*Y*=3E−6X+0.6951	0.9941	2.5–10.0
Naphthalene	*Y*=3E−6X+0.3251	0.9889	2.5–10.0
Indole	*Y*=6E−6X−1.3309	0.9912	5.0–20.0
*cis*-Jasmone	*Y*=1E−5X−0.3410	0.9988	12.5–50.0

a *Y* is the amount (μg kg^−1^) of volatile compound based on the peak area X generated using GC–MS with the defined program.

b Mixture of enantiomers of (3*S*)-*trans*-nerolidol and (3*R*)-*trans*-nerolidol, which were not separately quantified in this study.

**Table 2:** Sensory evaluation of the tea samples.

**Green tea sample**	**Aroma**		**Taste**		**Overall quality**
	Score	Characteristics	Score	Characteristics	
BAS-Pa	92.8±2.5 a⁎	slight herb-like, nut-like, roasty	89.8±3.2 a	more astringent and brisker	93.5±5.4 a
FUD-Pa	83.6±3.3 b	nut-like, green leafy note, roasty	81.7±2.7 b	brisk, astringent	81.3±4.4 b

⁎ Values with the same letter did not have significant difference between the same columns, using *t*-test.

**Table 3:** Volatiles with no significant differences in abundance (μg kg^−1^ DW) between ‘BAS’ and ‘FUD’ among the different infusions of processed green teas or fresh leaves (Fr).

**No.**	**Volatile compounds**	**BAS-Pa**	**FUD-Pa**	**–**
14	*cis*-citral	0.84±0.24	0.57±0.28	
18	Geranyl acetone	0.65±0.08	0.61±0.09	
23	α-Calacorene	Trace	ND	
27	Copaene	1.32±0.04	1.21±0.36	
34	Butyl butanoate	Trace	ND	
35	*cis*-3-Hexenyl hexanoate	1.48±0.17	1.64±0.36	
39	*cis*-3-Hexenyl acetate	3.01±0.01	3.24±0.10	
45	Hexadecane	0.78±0.31	ND	
46	Hentriacontane	ND	Trace	
47	Pentacosane	ND	Trace	
49	Hexacosane	ND	Trace	
50	Heptadecane	ND	Trace	
**No.**	**Volatile compounds**	**BAS-Fr**	**FUD-Fr**	–
9	Neo-allo-ocimene	3.17±0.28	2.56±0.06	
14	*cis*-citral	1.50±0.22	1.06±0.11	
34	Butyl butanoate	1.11±0.22	1. 56±0.67	
36	*cis*-3-Hexenyl-*trans*-2-hexenoate	8.78±2.39	7.89±1.78	
45	Hexadecane	0.94±0.16	2.17±1.61	
47	Pentacosane	1.28±0.67	0.94±0.37	
48	Heptacosane	ND	Trace	
49	Hexacosane	Trace	ND	
57	unknown	3.28±0.39	ND	
58	unkonwn	5.44±0.28	ND	
**No.**	**Volatile compounds**	**BAS-St**	**BAS-Pa**	**BAS-Fr**
3	*trans*-β-Ocimene	ND	ND	5.06±0.28
9	Neo-allo-ocimene	ND	ND	3.17±0.28
28	Farnesene	ND	ND	3.06±1.44
**No.**	**Volatile compounds**	**BAS-St**	**BAS-Pa**	**BAS-Fr**
36	*cis*-3-Hexenyl-*trans*-2-hexenoate	ND	ND	8.78±2.39
37	*trans*-2-Hexenyl butanoate	ND	ND	27.87±5.61
41	*trans*-2-Hexenal	ND	ND	2.67±0.17
45	Hexadecane	ND	Trace	Trace
47	Pentacosane	Trace	ND	Trace
49	Hexacosane	ND	ND	Trace
54	1-methyl-naphthalene	ND	ND	3.89±1.00
**No.**	**Compounds**	**FUD-St**	**FUD-Pa**	**FUD-Fr**
1	β-Myrcene	ND	ND	16.39±2.33
2	Limonene	ND	ND	10.39±0.83
3	*trans*-β-Ocimene	ND	ND	4.11±0.61
9	Neo-allo-ocimene	ND	ND	2.56±0.06
13	Nerol	ND	ND	5.39±0.83
16	Citral	ND	ND	7.83±1.06
36	*cis*-3-Hexenyl-*trans*-2-hexenoate	ND	ND	7.89±1.78
37	*trans*-2-Hexenyl butanoate	ND	ND	6.94±0.33
41	*trans*-2-Hexenal	ND	ND	9.00±1.94
45	Hexadecane	ND	ND	Trace
46	Hentriacontane	Trace	Trace	ND
47	Pentacosane	Trace	Trace	Trace
48	Heptacosane	Trace	ND	Trace
49	Hexacosane	ND	Trace	ND
50	Heptadecane	Trace	Trace	ND
54	1-Methyl-naphthalene	ND	ND	2.11±0.22

Note: The volatile compounds were putatively identified using NIST database and quantified based on internal reference compounds. DW-dry weight. ND-not detected.

**Table 4:** The most important compounds for observed variance in volatile profiles of pan-fire processed green teas between the two cultivars ‘BAS’ and ‘FUD’.

**No.**	**Compounds**	**VIP**	**No.**	**Compounds**	**VIP**
1	Linalool oxide I	1.323	9	β-Ocimene	1.231
2	Linaloloxide II	1.314	10	*cis*-3-Hexenyl isovalerate	1.228
3	Naphthalene	1.291	11	Unknown	1.224
4	Limonene	1.284	12	Geraniol	1.217
5	Citral	1.274	13	unknown	1.196
6	(+)-δ-Cadinene	1.255	14	Butyl butanoate	1.185
7	Methyl salicylate	1.246	15	Hotrienol	1.167
8	Methyl 2-methylvalerate	1.245			

**Table 5:** Threshold values and odor characteristics of detected volatiles.

**No.**	**Compounds**	**Threshold value (ppb)**	**Aroma quality**	**References**
1	β-Myrcene	4.9	Herbaceous, woody	www.leffingwell.com/odorthre.htm
2	Limonene	10.0	Citrus, terpenic	www.leffingwell.com/odorthre.htm
3	*trans*-β-Ocimene	340.0	Green, terpenic	www.leffingwell.com/odorthre.htm
4	β-Ocimene	34.0	Sweet	www.leffingwell.com/odorthre.htm
5	Linalool oxide I	6.0	Floral green	[2]
6	Linalool oxide II	6.0	Fruity	[2]
7	Linalool	0.8	Floral, fruity	[3]
8	Hotrienol	110.0	Ginger like	[4]
10	Epoxylinalol	6.0	Sweet, woody	[2]
11	α-Terpineol	330.0	Floral, sweet	[2]
13	Nerol	300.0	Rose, lime	[2]
15	Geraniol	3.2	Sweet floral	[4]
16	Citral	30.0	Citrus, lemon	www.leffingwell.com/odorthre.htm
18	Geranyl acetone	60.0	Fresh, rosy	www.leffingwell.com/odorthre.htm
22	β-Ionone	0.007	Dry, floral, fruity	[4]
24	*trans*-Nerolidol	2250.0	Floral, woody	www.leffingwell.com/odorthre.htm
32	Methyl salicylate	40.0	Wintergreen like	[2]
34	Butyl butanoate	100.0	Rotten apple	www.leffingwell.com/odorthre.htm
39	*cis*-3-Hexenyl acetate	31.0	Green; banana-like	www.leffingwell.com/odorthre.htm
40	Benzene-acetaldehyde	4.0	Green	www.leffingwell.com/odorthre.htm
41	*trans*-2-Hexenal	17.0	Green apple-like, bitter almond-like	www.leffingwell.com/odorthre.htm
42	Nonanal	1.0	Fatty, citrus, green	www.leffingwell.com/odorthre.htm
43	Heptanal	3.0	Fatty green	www.leffingwell.com/odorthre.htm
44	Decanal	2.0	citrus	www.leffingwell.com/odorthre.htm
51	*cis*-3-Hexen-1-ol	13.0	Lettuce-like	[4]
52	3-Octen-1-ol	1.0	Green, meaty	[2]
53	Naphthalene	5.0	naphthalene	[5]
55	Indole	1.0	Faecal, animal-like	[6]
56	*cis*-Jasmone	1.9	Floral, jasmine-like	This study

**Table 6:** Volatiles that were present in the fresh leaf sample infusions but not detected among the processed green tea infusions of ׳BAS׳ and ׳FUD׳.

**Volatile compounds**	**BAS-St**	**BAS-Pa**	**BAS-Fr**
*trans*-β-Ocimene	ND	ND	5.06±0.28
Neo-allo-ocimene	ND	ND	3.17±0.28
Farnesene	ND	ND	3.06±1.44
*cis*-3-Hexenyl-trans-2-hexenoate	ND	ND	8.78±2.39
*trans*-2-Hexenyl butanoate	ND	ND	27.87±5.61
*trans*-2-Hexenal	ND	ND	2.67±0.17
1-methyl-Naphthalene	ND	ND	3.89±1.00
**Compounds**	**FUD-St**	**FUD-Pa**	**FUD-Fr**
β-Myrcene	ND	ND	16.39±2.33
Limonene	ND	ND	10.39±0.83
*trans*-β-Ocimene	ND	ND	4.11±0.61
Neo-allo-ocimene	ND	ND	2.56±0.06
Nerol	ND	ND	5.39±0.83
Citral	ND	ND	7.83±1.06
*cis*-3-Hexenyl-*trans*-2-hexenoate	ND	ND	7.89±1.78
*trans*-2-Hexenyl butanoate	ND	ND	6.94±0.33
*trans*-2-Hexenal	ND	ND	9.00±1.94
1-methyl-Naphthalene	ND	ND	2.11±0.22

Note: Abundances of volatiles were presented as μg kg^−1^ DW. ND-not detected.
