# Supplementary material for: Data on green tea flavor determinantes as affected by cultivars and manufacturing processes
Source: Data Brief. 2016 Dec 21;10:492–8. doi: 10.1016/j.dib.2016.12.025 (PMC5196232; doi:10.1016/j.dib.2016.12.025)
Supplement: Supplementary file 1 — Supplementary material [file mmc2.docx]

*Data article*

**Data on green tea flavor determinates as affected by cultivars and manufacturing processes**

Zhuo-Xiao Han^a^, Mohammad M. Rana^a,b^, Guo-Feng Liu^a^, Ming-Jun Gao^c^, Da-Xiang Li^a^, Fu-GuangWu^d^, Xin-Bao Li^d^, Xiao-Chun Wan^a^, Shu Wei^a^*

**Authors’ Declaration of interest**

'Conflicts of interest: none'.
